# Up-modulation of PLC-β2 reduces the number and malignancy of triple-negative breast tumor cells with a CD133^+^/EpCAM^+^ phenotype: a promising target for preventing progression of TNBC

**DOI:** 10.1186/s12885-017-3592-y

**Published:** 2017-09-04

**Authors:** Federica Brugnoli, Silvia Grassilli, Paola Lanuti, Marco Marchisio, Yasamin Al-Qassab, Federica Vezzali, Silvano Capitani, Valeria Bertagnolo

**Affiliations:** 1 Signal Transduction Unit, Division of Anatomy and Histology, Department of Morphology, Surgery and Experimental Medicine, University of Ferrara, Via Fossato di Mortara, 70, 44121 Ferrara, Italy; 2 Department of Medicine and Aging Science, “G. d’Annunzio” University of Chieti-Pescara, Chieti, Italy; 3 Center of Aging Sciences and Translational Medicine (CeSI-MeT), “G. d’Annunzio” University of Chieti-Pescara, Chieti, Italy; 4 College of Medicine, Department of Anatomy, University of Baghdad, Baghdad, Iraq; 5 LTTA Centre, University of Ferrara, Ferrara, Italy

**Keywords:** Triple-negative breast cancer (TNBC), CD133, EpCAM, PLC-β2, Breast cancer stem cell (BCSC), Proliferation, Invasiveness

## Abstract

**Background:**

The malignant potential of triple negative breast cancer (TNBC) is also dependent on a sub-population of cells with a stem-like phenotype. Among the cancer stem cell markers, CD133 and EpCAM strongly correlate with breast tumor aggressiveness, suggesting that simultaneous targeting of the two surface antigens may be beneficial in treatment of TNBC. Since in TNBC-derived cells we demonstrated that PLC-β2 induces the conversion of CD133^high^ to CD133^low^ cells, here we explored its possible role in down-modulating the expression of both CD133 and EpCAM and, ultimately, in reducing the number of TNBC cells with a stem-like phenotype.

**Methods:**

A magnetic step-by-step cell isolation with antibodies directed against CD133 and/or EpCAM was performed on the TNBC-derived MDA-MB-231 cell line. In the same cell model, PLC-β2 was over-expressed or down-modulated and cell proliferation and invasion capability were evaluated by Real-time cell assays. The surface expression of CD133, EpCAM and CD44 in the different experimental conditions were measured by multi-color flow cytometry immunophenotyping.

**Results:**

A CD133^+^/EpCAM^+^ sub-population with high proliferation rate and invasion capability is present in the MDA-MB-231 cell line. Over-expression of PLC-β2 in CD133^+^/EpCAM^+^ cells reduced the surface expression of both CD133 and EpCAM, as well as proliferation and invasion capability of this cellular subset. On the other hand, the up-modulation of PLC-β2 in the whole MDA-MB-231 cell population reduced the number of cells with a CD44^+^/CD133^+^/EpCAM^+^ stem-like phenotype.

**Conclusions:**

Since selective targeting of the cells with the highest aggressive potential may have a great clinical importance for TNBC, the up-modulation of PLC-β2, reducing the number of cells with a stem-like phenotype, may be a promising goal for novel therapies aimed to prevent the progression of aggressive breast tumors.

**Electronic supplementary material:**

The online version of this article (10.1186/s12885-017-3592-y) contains supplementary material, which is available to authorized users.

## Background

Triple-negative breast cancer (TNBC), which accounts for 10% to 24% of invasive breast cancers, is typically a high-grade tumor with a great propensity to metastasize [1]. Different studies grouped TNBC on the basis of immunophenotype and of RNA and DNA genomic profiles, identifying subtypes with variable potentiality of aggressiveness. In all studies, the more aggressive subtypes were those associated with the expression of immunomodulatory and stem-like molecules [2, 3]. In particular, the ability of TNBC cells to proliferate, progress, and spread is also based on a limited sub-population of cells with properties similar to stem cells, defined as “breast cancer stem cells” (BCSCs) [4]. Several stemness markers have been described to identify BCSCs, such as CD44, CD24, CD133, EpCAM, CD166, Lgr5, CD47, ALDH1, and ABCG2 [5, 6]. Since their expression profiles showed a large variability within breast cancer subtypes, especially for TNBCs, none of them may be singly correlated with prognosis or with specific therapies of TNBC patients [3, 5]. It is then undoubted that the simultaneous targeting of various markers expressed on BCSCs may have advantage in the treatment of highly aggressive breast tumors. In this context, CD133 and EpCAM are highly promising target antigens since, beyond to be markers of BCSCs, they have a direct relationship with malignancy of breast tumors. In particular, CD133 expression in breast cancer significantly correlates with tumor stage, tumor size, occurrence of lymph node metastases and sensitivity to neoadjuvant chemotherapy [7, 8]. In TNBC, CD133^+^ cells with cancer stem cell characteristics associate with vasculogenic mimicry [9]. The recent use of CD133 to detect circulating tumor cells in TNBC patients [10] has increased attention to this marker highlighting its role in establishing prognostic and predictive value in TNBCs. Concerning EpCAM, its over-expression was observed in up to 70% of breast tumors in which it strongly correlates with a higher risk of recurrence [11]. The use of EpCAM as a marker for detecting disseminated breast cancer cells in bone marrow strongly suggests that EpCAM^+^ breast cancer cells possess an enhanced ability to metastasize [12]. Nevertheless, the sole over-expression of CD133 or EpCAM in TNBC was correlated with poorer prognosis in about 60% of tumors [13, 14]. This evidence, on one hand, indicates that the selective removal of CD133^+^ or EpCAM^+^ cells may not be sufficient to eradicate cells with the most aggressive phenotype, like cancer stem cells, and on the other hand that the simultaneous targeting of the two surface antigens may be of clinical relevance in treatment of TNBC patients. In recent years, a toxin-based system to simultaneously target CD133 and EpCAM in the same cell was developed in different carcinoma models, including inflammatory breast carcinoma, showing a potent inhibition of proliferation in vitro and the regression of HNSCC (Head and neck squamous cell carcinoma) in vivo [15]. Despite these encouraging results, the use on human tumors is far for being advantageous, due to the high costs of toxin generation and, more importantly, to the off-target effects or to the generation of anti-toxin antibodies having adverse effects against extended treatments [16].

In breast tumor-derived cells with different phenotypes, we demonstrated that the expression of CD133 is strongly correlated to the levels of the beta2 isoform of the phosphoinositide-dependent phospholipase C (PLC-β2) [17, 18], ectopically expressed in the large majority of primary invasive mammary tumors of all histological subtypes [19]. Consistently, we also found that in both MDA-MB-231 and MDA-MB-468 cells, showing a TNBC basal-B and a basal-A phenotype, respectively [20], the over-expression of PLC-β2 induced the conversion of CD133^high^ to CD133^low^ cells [17].

Here we explored the possible role of PLC-β2 in modulating the expression of both CD133 and EpCAM in triple-negative breast tumor cells, in order to assess if strategies aimed to up-modulate this PLC isozyme may be useful in reducing the expression of these BCSCs markers and, eventually, in reducing the number of cells with a stem-like phenotype.

## Methods

All reagents were from Sigma (St Louis, MO) unless otherwise indicated.

### Cell culture

The breast cancer-derived cell lines MDA-MB-231 (HTB-26), MDA-MB-468 (HTB-132) and MCF7 (HTB-22) were purchased from the American Type Culture Collection (Rockville, MD) and grown in Dulbecco’s modified Eagle’s medium (DMEM, Gibco Laboratories, Grand Island, NY) supplemented with 10% fetal bovine serum (FBS, Gibco Laboratories), at 37 °C in a humidified atmosphere of 5% CO_2_ in air. Cells were monthly tested for mycoplasm and other contaminations and quarterly subjected to cell identification by single-nucleotide polymorphism. Cell viability was determined with a hemocytometer-based Trypan blue dye exclusion cell quantification.

### Immunophenotyping

The expression of CD133, EpCAM and CD44 surface antigens were evaluated by flow cytometry following a previously described procedure [21]. In a one-tube assay, cells were stained with phycoerythrin (PE)-conjugated anti-CD133/2 (293C3) and fluorescein isothiocyanate (FITC)-conjugated anti-CD326 (EpCAM) (Miltenyi Biotec, Bologna, I) or with PE-conjugated anti-CD133, FITC-conjugated anti-EpCAM and allophycocyanin (APC)-conjugated anti-CD44 (Becton Dickinson, San José, CA) monoclonal antibodies. All samples were analyzed by a FACSCalibur flow cytometer (Becton Dickinson) with the CellQuest Pro 6.0 software (Becton Dickinson). 20,000 non-debris events in the morphological gate were recorded for each sample. All antibodies were titrated under assay conditions and optimal photomultiplier (PMT) gains were established for each channel, as previously reported [22].

Data were analysed using FlowJo™ software (TreeStar, Ashland, OR) and reported as percentage of positive cells or as mean fluorescence intensity (MFI) values.

### Magnetic step-by-step cell isolation

MDA-MB-231 sub-populations enriched in CD133^+^ and/or EpCAM^+^ cells were obtained by means of the MACS immunomagnetic separation system, essentially as described by Pierzchalski et al. [23]. In particular, cells were firstly labeled with CD133/1 MicroBeads (Miltenyi Biotech) and subjected to positive magnetic cell separation through MACS SD columns in the field of the MACS magnet (Miltenyi Biotech), according to manufacturer’s instructions. Both CD133^−^ and CD133^+^ cells were then subjected to a second positive selection after magnetic labelling with CD326 MicroBeads (Miltenyi Biotech). The obtained CD133^−^/EpCAM^−^, CD133^−^/EpCAM^+^, CD133^+^/EpCAM^−^ and CD133^+^/EpCAM^+^ enriched sub-populations were cultured in the above reported medium and subjected to cytofluorimetrical analysis, to invasion assays and to modulation of PLC-β2 expression.

### Cell proliferation and invasion assays

Proliferation and invasiveness of cellular subsets derived from magnetic separation were determined by means of the xCELLigence Real-Time Cell Analyzer System (RTCA, Acea Bioscences Inc., San Diego, CA), developed to monitor cell events in real time by measuring the electrical impedance produced by cells, as previously reported [17]. In particular, to measure cell proliferation, 5000 cells/well were plated and signal detection was enabled every 15 min up to 96 h. For the invasion assay, 40,000 cells/well were seeded onto the top chambers covered with a layer of Matrigel (Becton Dickinson) diluted 1:20. The bottom chambers were filled with medium containing 10% serum and the signal was detected every 15 min for a total of 24 h.

Cell invasion was also evaluated by means of Boyden Chamber assays according to the protocol recommended from Chemicon (Tamecula, CA). The Cell Invasion Assay Kit (ECM550) is made up of invasion chambers containing inserts with an 8 μm pore size polycarbonate membrane over which a thin layer of ECMatrix™ solution was applied, following a procedure previously reported [24].

### Immunofluorescence analysis

Cellular populations derived from magnetic separation were grown onto glass slides, fixed with freshly prepared 4% paraformaldehyde, washed in PBS and reacted with the anti-PLC-β2 (#SC-206, Santa Cruz Biotechnology, Santa Cruz, CA) antibody in a Net Gel solution, alone or in combination with the anti-CD133 (W6B3C1, Miltenyi Biotech) or with the anti-EpCAM (NCL-ESA, Leica Biosystems, Buccinasco, I) antibodies, respectively, following a previously reported procedure [18]. Samples were then incubated with a FITC and/or TRITC conjugated secondary antibody and, after washes, with 0.5 mg/ml 4',6-diamidino-2-phenylindole (DAPI), dried with ethanol and mounted in glycerol containing 1,4-diazabicyclo [2.2.2] octane (DABCO) to retard fading. Fluorescent samples were observed with a Nikon Eclipse TE2000-E microscope (Nikon), acquiring cell images by the ACT-1 software for a DXM1200F digital camera (Nikon ﻿S.p.a., Florence, I). To measure PLC-β2 staining, digitized images were analyzed with the ImageJ software, following the manufacturer’s instructions (http://rsb.info.nih.gov/ij/).

### Modulation of PLC-β2 expression

PLC-β2 over-expression was performed by transient transfection with a plasmid expressing a full-length human PLC-β2 and the specific down-modulation of the PLC was achieved by using specific siRNAs (Santa Cruz Biotechnology), following previously described procedures [18]. An empty vector and a non-silencing scramble siRNAs, respectively, were used as negative controls. The transfected cells were incubated at 37 °C in a 5% CO_2_ atmosphere for 48 h then subjected to RTCA and to cytofluorimetrical analysis.

### Statistical analysis

Statistical analysis was performed by using the two-tailed Student’s t-test for unpaired data. *P* values <0.05 were considered statistically significant.

## Results

### A MDA-MB-231 sub-population expressing high surface levels of CD133 and EpCAM shows elevated proliferation and invasion capability

**Fig. 1:**
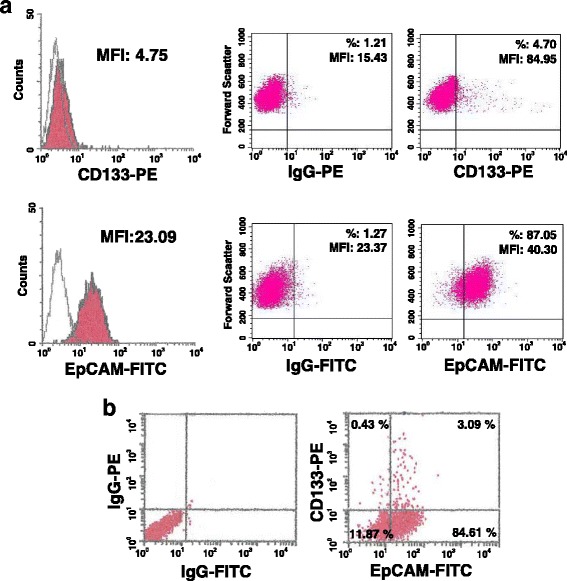
Expression of CD133 and EpCAM in MDA-MB-231 cells. In **a** representative cytofluorimetrical evaluation of CD133 and EpCAM surface levels in MDA-MB-231 cells after labelling with a PE-conjugated anti-CD133 antibody or with a FITC-conjugated anti-EpCAM antibody. The expression of each antigen is shown, on the left, on a frequency distribution histogram (count vs. PE or FITC signal) in which the mean fluorescence intensity (MFI) of the entire population is reported. The red filled histograms represent positive staining for CD133 or EpCAM and the open histograms, outlined by gray lines, show staining with isotype matched antibodies. On the right, surface expression of each antigen is shown on a biparametric dot plot and the percentage and MFI of positive cells are indicated. In **b** representative surface expression of both CD133 and EpCAM in MDA-MB-231 cells after double labelling with a PE-conjugated anti-CD133 and with a FITC-conjugated anti-EpCAM antibodies is shown on a biparametric dot plot and the percentage of cells in all the derived quadrants is indicated

By means of a cytofluorimetrical approach, we confirmed the existence of cells expressing CD133 at surface level in the highly tumorigenic TNBC derived MDA-MB-231 cell line and we revealed that almost 90% of cells result EpCAM^+^ (Fig. 1a). As expected [14, 25], the mean expression level of EpCAM in MDA-MB-231 cell, showing a mesenchymal-like phenotype (basal-B TNBC), is definitely lower than that of MCF7 cells, sharing a luminal B phenotype and low invasive potential, and of MDA-MB-468, a TNBC derived cell line with an epithelial-like phenotype (basal-A TNBC) and moderately invasive, 100% expressing high levels of CD133 (Additional file 1: Fig. S1A, B).

The contemporary use of the anti-CD133 and anti-EpCAM antibodies showed the presence of MDA-MB-231 cells expressing different levels of the two antigens at surface levels and allowed to identify a CD133^+^/EpCAM^+^ sub-population, accounting for about 3% of cells (Fig. 1b).

**Fig. 2:**
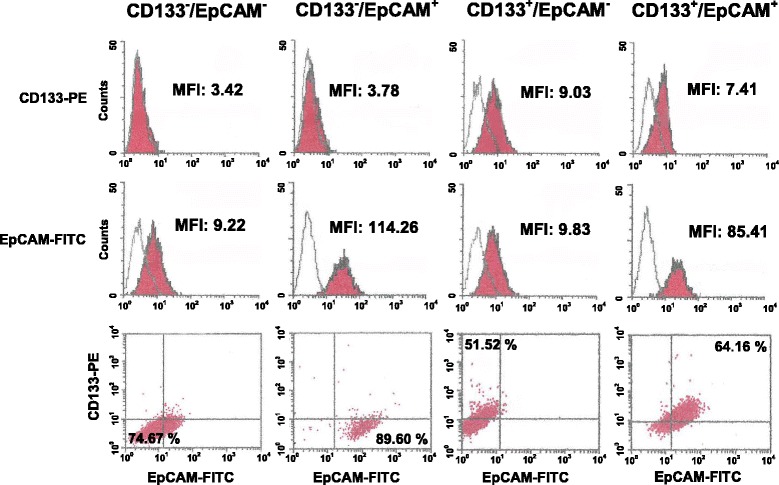
CD133 and EpCAM surface levels in MDA-MB-231 sub-populations. MDA-MB-231 cells were subjected to positive immunomagnetic separation after labeling with MicroBeads-conjugated anti-CD133 antibody followed by the positive selection through column of cells labeled with MicroBeads-conjugated anti-EpCAM antibody. Surface levels of CD133 and EpCAM were evaluated in all sub-populations after double labelling with a PE-conjugated anti-CD133 and with a FITC-conjugated anti-EpCAM antibody. The expression of each antigen is shown on a frequency distribution histogram (count vs. PE or FITC signal) in which the MFI of the entire population is reported. The red filled histograms represent positive staining for CD133 or EpCAM and the open histograms, outlined by gray lines, show staining with matched isotype antibodies. The dot plot analysis was used to assess the enrichment in CD133^−^/EpCAM^−^, CD133^−^/EpCAM^+^, CD133^+^/EpCAM^−^ and CD133^+^/EpCAM^+^ cells in all subsets and the percentage of the main cell phenotype in each enriched sub-population is indicated. The data are indicative of three separate experiments

At variance with hepatocellular carcinoma (HCC), in which the features of cells with different CD133/EpCAM phenotypes were subjected to both in vitro and in vivo characterization [26], no information is available on TNBC derived cells showing variable surface levels of the two antigens. In order to study the correlation of CD133 and/or EpCAM with malignant features of MDA-MB-231, a magnetic step-by-step cell isolation with antibodies directed against the two surface antigens was performed. Since CD133^+^ cells are rare elements in the MDA-MB-231 cell population, we applied the MACS technique instead of the currently used Fluorescence-Activated Cell Sorting, thus ensuring the achievement of a relatively high number of cells in a short time [17, 23]. We obtained populations enriched in CD133^−^/EpCAM^−^, CD133^−^/EpCAM^+^, CD133^+^/EpCAM^−^ or CD133^+^/EpCAM^+^ cells (Fig. 2). In particular, both CD133^−^/EpCAM^+^ and CD133^+^/EpCAM^+^ sub-populations showed a relatively high mean expression level of EpCAM, indicating that the applied isolation procedure selected the cells with the higher surface levels of this adhesion molecule (Fig. 2).

All sub-populations, grown in the same standard conditions, showed viability comparable to control cells and stable CD133 and EpCAM expression levels up to at least 3 passages in monolayer culture (data not shown).

**Fig. 3:**
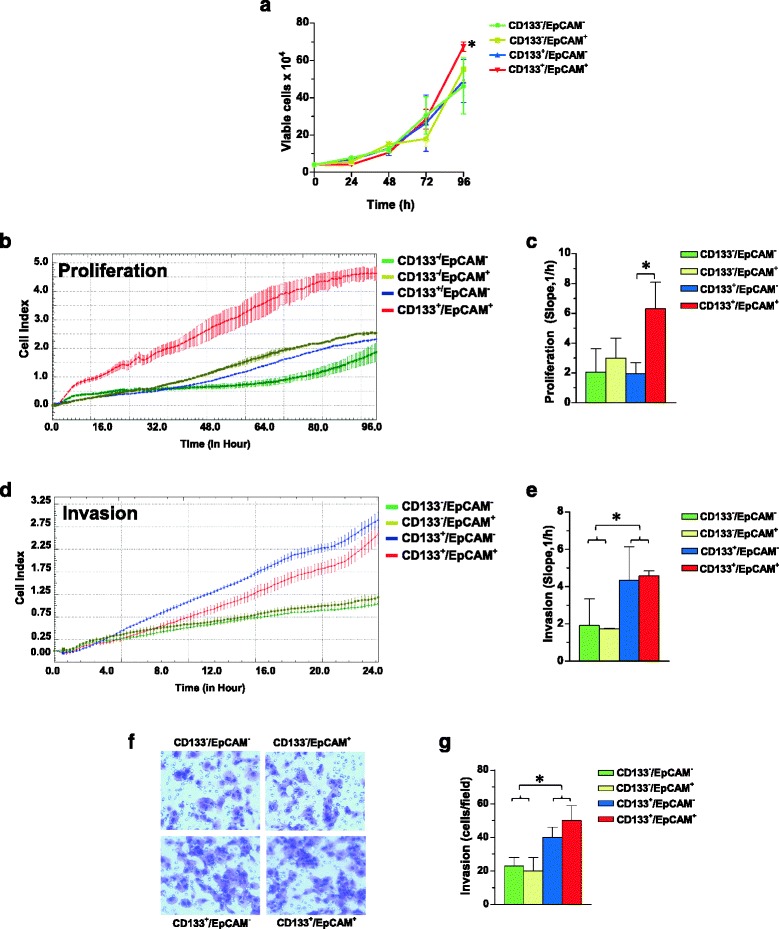
CD133 and EpCAM related proliferation and invasion capability of MDA-MB-231 sub-populations. Cells derived from immunomagnetic separations were immediately grown in culture medium for 96 h and daily counted by hemocytometer after Trypan Blue staining (**a**). After 24 h from separation, the cellular subsets were subjected to dynamic monitoring of proliferation (**b**) and invasion through Matrigel (**d**) using the xCELLigence RTCA system. Cell Index (CI) is reported and error bars indicate ±SD. The correspondent Slope analysis, that describes the steepness, incline, gradient, and changing rate of the CI curves over time, is shown in **c** and **e** respectively. In **f** a representative image of ECM invading cells in a Boyden Chamber assay, whose number is reported in **g**. The data are the mean of three separate experiments ± SD. The asterisks indicate statistically significant differences (*P* < 0.05)

Since we previously demonstrated that CD133^high^ TNBC derived cells exhibit low proliferation rate but high invasion capability through Matrigel [17] and owing to the evidence that specific down-modulation of EpCAM decreases proliferation in the MDA-MB-231 cell line [25, 27] all sub-populations were subjected to analysis of proliferation and invasiveness. As shown in Fig. 3, the CD133^+^/EpCAM^+^ enriched cell population revealed the highest proliferation rate, measured in cells directly derived from magnetic separation (Fig. 3a) as well as in cells that, after 24 h from separation, were subjected to the xCELLigence assay (Fig. 3b, c).

Concerning invasion aptitude, both the xCELLigence strategy (Fig. 3d, e) and the classical ECM invasion assay (Fig. 3f, g) revealed for the two CD133^+^ enriched sub-populations a significantly higher capability to pass through Matrigel, consistent with our previous results obtained in the same cell line [17]. At variance, no difference in invasion capability were correlated with the expression levels of EpCAM in the different sub-populations (Fig. 3d-g).

### PLC-β2 down-modulates the expression of both CD133 and EpCAM in the CD133^+^/EpCAM^+^ MDA-MB-231 sub-population.

**Fig. 4:**
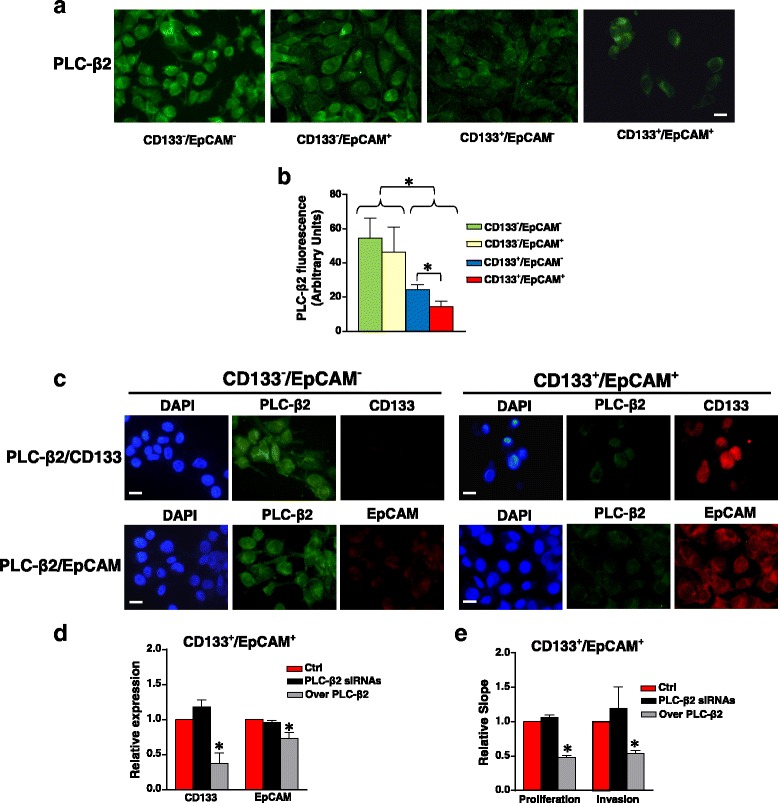
PLC-β2-related features in sub-populations enriched in CD133^+^ and/or EpCAM^+^ cells. In **a** representative fluorescence microscopy images of MDA-MB-231 sub-populations enriched in CD133^−^/EpCAM^−^, CD133^−^/EpCAM^+^, CD133^+^/EpCAM^−^ and CD133^+^/EpCAM^+^ cells subjected to immunocytochemical analysis with the anti-PLC-β2 antibody. The fluorescence intensity of PLC-β2 staining was calculated in digitized images by the ImageJ software and reported in **b** as arbitrary units. In **c** immunocytochemical analysis of the indicated sub-populations after simultaneous staining with the anti-PLC-β2 antibody (green fluorescence) and with the anti-CD133 or anti- EpCAM antibody (red fluorescence). In **d** the enriched CD133^+^/EpCAM^+^ sub-population was transfected with siRNAs specific for PLC-β2 (PLC-β2 siRNAs) or with a construct expressing the human PLC-β2 (Over PLC-β2) and subjected to simultaneous flow cytometry analysis of CD133 and EpCAM surface expression. Non-silencing scramble siRNAs or an empty vector were used as controls (Ctrl). In each experimental condition, fold change is compared with Ctrl, taken as 1. Proliferation and invasiveness of cells in the same experimental conditions were measured by the xCELLigence system (**e**). All the data are the mean of three separate experiments performed in triplicate ± SD. *P < 0.05. Bar = 20 μm

In breast tumor derived cell lines with different phenotypes, including MDA-MB-231, we previously found that the level of CD133 inversely correlates with that of PLC-β2 [17]. By means of immunocytochemical analysis, here we confirmed that the two purified CD133^−^ cellular subsets contain a PLC-β2 amount significantly higher than the CD133^+^ enriched sub-populations (Fig. 4a, b). While the PLC-β2 level in CD133^−^ cells appeared unrelated to EpCAM expression, cells expressing high levels of the two antigens at surface level showed the lowest PLC-β2 (Fig. 4a, b). A double staining of PLC-β2 and EpCAM or CD133 was performed on the CD133^−^/EpCAM^−^ and CD133^+^/EpCAM^+^ subsets and the immunofluorescence analysis showed that populations are homogeneous in terms of PLC-β2 expression and in terms of its relationship with the surface antigens (Fig. 4c).

As in both MDA-MB-231 and MDA-MB-468 we previously found that the over-expression of PLC-β2 induced the CD133^high^ to CD133^low^ conversion [17], the role of the PLC isozyme in modifying CD133 and/or EpCAM was investigated. PLC-β2 was then over-expressed in the CD133^+^/EpCAM^+^ enriched sub-population, which shows the lowest level of the protein, demonstrating a significant decrease of the surface expression of both antigens (Fig. 4d), in parallel with reduced proliferation and invasion aptitude (Fig. 4e).

### PLC-β2 down-modulates MDA-MB-231 sub-populations with a stem-like phenotype

**Fig. 5:**
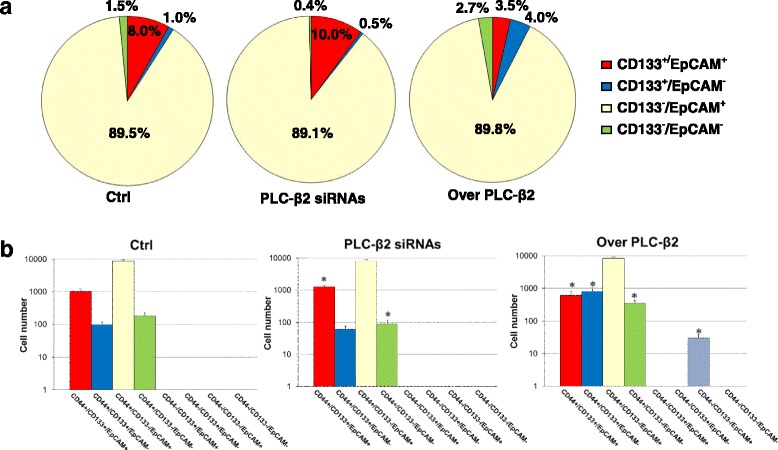
Relationship between PLC-β2 and MDA-MB-231 sub-populations with a stem-like phenotype. In **a** MDA-MB-231 cells transfected with siRNAs specific for PLC-β2 (PLC-β2 siRNAs) or with a construct expressing human PLC-β2 (Over PLC-β2) were subjected to a bi-parametric flow cytometry by direct staining with the anti-CD133 and anti-EpCAM fluorescent antibodies to evaluate the number of CD133^−^/EpCAM^−^, CD133^−^/EpCAM^+^, CD133^+^/EpCAM^−^ and CD133^+^/EpCAM^+^ cells. Non-silencing scramble siRNAs or an empty vector were used as control (Ctrl). The data, representative of three separate experiments, are presented on pie charts and the percentage of each cellular subset is reported. In **b** MDA-MB-231 cells in which PLC-β2 was forcedly silenced (PLC-β2 siRNAs) or over-expressed (Over PLC-β2) were simultaneously stained with APC-conjugated anti-CD44, FITC-conjugated anti-EpCAM and PE-conjugated anti-CD133 and subjected to flow cytometry evaluation of CD44, EpCAM and CD133 surface levels. Data are the mean of three separate experiments performed in triplicate ± SD. *P < 0.05

Once established that PLC-β2 may affect the levels of both CD133 and EpCAM in TNBC derived cellular subsets, we investigated its possible role in affecting, in the entire population, the number of cells expressing the two surface antigens. A cytofluorimetrical analysis was then performed on MDA-MB-231 cells in which PLC-β2 was forcedly up-modulated, demonstrating a substantial decrease of the CD133^+^/EpCAM^+^ sub-population and the concomitant increase of CD133^−^/EpCAM^−^ and CD133^+^/EpCAM^−^ cells (Fig. 5a, b). Accordingly, an opposite effect was obtained by silencing PLC-β2 (Fig. 5a, b).

To better investigate the role of PLC-β2 in modulating the phenotype of TNBC cells, a cytofluorimetrical analysis after the contemporary staining with the anti-CD44, anti-CD133 and anti-EpCAM antibodies was performed in MDA-MB-231 cells in which the protein was forcedly modulated. As shown in Fig. 5b, no CD44^−^ cells were detected in the wild-type population and in cells in which the PLC was silenced. On the other hand, the over-expression of PLC-β2 reduced the number of cells with a CD44^+^/CD133^+^/EpCAM^+^ phenotype (Fig. 5b) and induced the appearance of a small population of CD44−/CD133−/EpCAM+ cells (Fig. 5b).

## Discussion

Unlike other subtypes of breast carcinomas, TNBC lacks a specific targeted therapy and its tumor heterogeneity requires subclass-dependent treatments, often not sufficient to avoid worse prognosis [1, 2]. BCSCs have been suggested to contribute to the increased aggressiveness and poor prognosis of TNBC. However, the high intratumor stemness heterogeneity reduces the prognostic and therapeutic value of the presence of tumor-initiating cells in this breast tumor subtype [28]. Among the stemness markers in TNBC, CD133 and EpCAM may be of interest since they have a direct relationship with malignancy of breast tumors. In particular, TNBC derived cells expressing high levels of CD133 show larger adhesion area and lower proliferation rate, indicative of a less undifferentiated phenotype, but higher invasive capability and increased expression of proteins involved in metastasis and drug-resistance of breast tumors [17]. Epigenetic changes (DNA methylation, acetylation, chromatin modification, microRNA, etc.) have also been correlated with CD133 in TNBC breast cancer stem cells, possibly related to the nuclear localization of this glycoprotein [29]. On the other hand, the adhesion molecule EpCAM is generally expressed by TNBC at levels reflecting the different subtypes, being over-expressed in basal-A and very low or absent in basal-B subtypes [14]. In TNBC derived cells, EpCAM can significantly promote the proliferation [30] but its role in invasiveness seems to be strikingly correlated with the specific subtypes [31].

In lung cancer, cells over-expressing both CD133 and EpCAM are more abundant than in normal tissues [32] and, in HCC derived cells, the CD133+/EpCAM+ sub-population shows increased colony-formation ability, drug-resistance, more spheroid formation by cultured cells and stronger tumorigenicity in NOD/SCID mice [26]. Here we correlated the simultaneous expression of CD133 and EpCAM with malignancy of breast tumor cells revealing the unprecedented evidence that a CD133^+^/EpCAM^+^ sub-population with more aggressive potential is present in the MDA-MB-231 cell line, showing a basal-B TNBC phenotype [25]. In particular, we found that the CD133^+^/EpCAM^+^ enriched cell population showed high proliferation rate, confirming the role described for the adhesion molecule in regulating growth of TNBC derived cells [14] but also suggesting that the contemporary surface localization of CD133 and EpCAM identifies a subset of cells with enhanced in vitro growth rate. Concerning invasion capability, the CD133^+^ enriched sub-populations showed a significantly higher capability to pass through Matrigel, consistent with our previous results obtained in the same cell line [17]. We failed to correlate invasion capability with the different expression levels of EpCAM, according with the results obtained by over-expressing the adhesion molecule in this cell line [27]. Our in vitro results indicate that a CD133^+^/EpCAM^+^ population with high proliferation rate and invasive potential may have a role in progression of TNBC, as already hypothesized for HCC and lung cancer [26, 32].

In breast tumor derived cell lines with different phenotypes we previously found that the level of CD133 inversely correlates with PLC-β2 and that over-expression of this PLC isozyme in MDA-MB-231 cells down-modulates CD133 at both membrane and intracellular levels [17]. Here we confirmed that the CD133^−^ cellular subsets contain a PLC-β2 amount significantly higher than the CD133^+^ enriched sub-populations and we demonstrated that the concomitant presence of CD133 and EpCAM at surface level characterizes the MDA-MB-231 cells with the lowest PLC-β2 level. In addition, we found that the forced expression of PLC-β2 in the CD133^+^/EpCAM^+^ subset significantly reduced the surface expression of both antigens. A concomitant reduction of both proliferation and invasion was observed, confirming our previous results indicating that up-regulation of PLC-β2 in triple negative cells expressing high levels of CD133 reduces their invasion capability [17] but also suggesting that PLC-β2, down-modulating EpCAM, negatively affects proliferation of TNBC derived cells.

When PLC-β2 was forcedly modulated in the entire MDA-MB-231 population, the number of CD133^+^/EpCAM^+^ cells significantly decreased, allowing to conclude that, in TNBC derived cells, this PLC isozyme may regulate the size of a cellular subset with high malignant potential, in terms of proliferation, invasion capability and surface expression of tumor stem cell markers.

To better investigate the role of PLC-β2 in modulating the stem cell phenotype of TNBC cells, we also evaluated the surface expression of CD44, a well-described cancer stem cell marker in breast tumors and a negative prognosticator in TNBC [33]. The contemporary staining of MDA-MB-231 with anti-CD44, anti-CD133 and anti-EpCAM antibodies demonstrated that the over-expression of PLC-β2 reduces the number of CD44^+^/CD133^+^/EpCAM^+^ cells. This phenotype, if shared by cells purified from pancreatic cancer (PC), correlates with increased cell growth, migration, clonogenicity, and self-renewal ability [34]. We also found that PLC-β2 induced the appearance of CD44^−^/EpCAM^+^ cells, usually found in normal breast cells [35], allowing to deduce that, at least in basal-B TNBC derived cells, high levels of this PLC isozyme may revert the malignant phenotype of a small number of cells, thus inducing a “normal-like” phenotype. This is also in agreement with our previous results obtained in low invasive breast tumor derived cells, in which high levels of PLC-β2 characterize cells with an epithelial-like phenotype and counteract the epithelial to mesenchymal shift induced by low oxygen availability [18]. Up-regulation of PLC-β2 in TNBC may then constitute a strategy to reduce the number of cells with high malignant potential that may take advantage of drugs already known to modulate this PLC isozyme [36].

## Conclusions

Overall, this work adds substantial information about the role of PLC-β2 in invasive breast tumors, demonstrating that its up-regulation in cells with a basal-B triple-negative phenotype is sufficient to down-modulate the expression of surface antigens crucial for malignancy and to reduce the number of cells with a stem-like phenotype. Given that, unlike other breast cancer subtypes, effective targeted therapy is not currently available for TNBC tumors and considering that the selective removal of BCSCs cells may have a great clinical importance, our results indicate that up-modulation of PLC-β2 is a promising tool for novel therapies aimed to prevent the progression of aggressive breast tumors.

## Additional file

Additional file 1: Figure S1. Surface CD133 and EpCAM, proliferation and invasiveness in breast derived cell lines. In **A**, representative cytofluorimetrical evaluation of CD133 and EpCAM surface levels in MCF7 and MDA-MB-468 cells after labelling with a PE-conjugated anti-CD133 antibody or with a FITC-conjugated anti-EpCAM antibody. The staining with isotype matched antibodies (IgG) is used as a control. The expression of each antigen is shown on a biparametric dot plot and the percentage and MFI of positive cells are indicated at the upper right of each panel. In **B**, MDA-MB-231, MCF7 and MDA-MB-468 cells were subjected to dynamic monitoring of proliferation and invasion through Matrigel using the xCELLigence RTCA system. Cell Index (CI) is reported and error bars indicate ±SD. The correspondent Slope analysis, that describes the steepness, incline, gradient, and changing rate of the CI curves over time, is shown on the right. The data were collected from three separate experiments (PDF 364 kb)

## Abbreviations

APC: Allophycocyanin
BCSC: Breast cancer stem cell
DAPI: 4′,6-diamidino-2-phenylindole
DMEM: Dulbecco’s Modified Eagle’s Medium
EpCAM: Epithelial cell adhesion molecule
FBS: Fetal bovine serum
FITC: Fluorescein isothiocyanate
MFI: Mean fluorescence intensity
PBS: Phosphate buffered-saline
PE: Phycoerythrin
PLC: Phospholipase C
PMT: Optimal photomultiplier
RTCA: Real-time cell assays
TNBC: Triple-negative breast cancer
