# Supplementary figures and images for: Up-modulation of PLC-β2 reduces the number and malignancy of triple-negative breast tumor cells with a CD133+/EpCAM+ phenotype: a promising target for preventing progression of TNBC
Source: BMC Cancer. 2017 Sep 4;17:617. doi: 10.1186/s12885-017-3592-y (PMC5584040; doi:10.1186/s12885-017-3592-y)

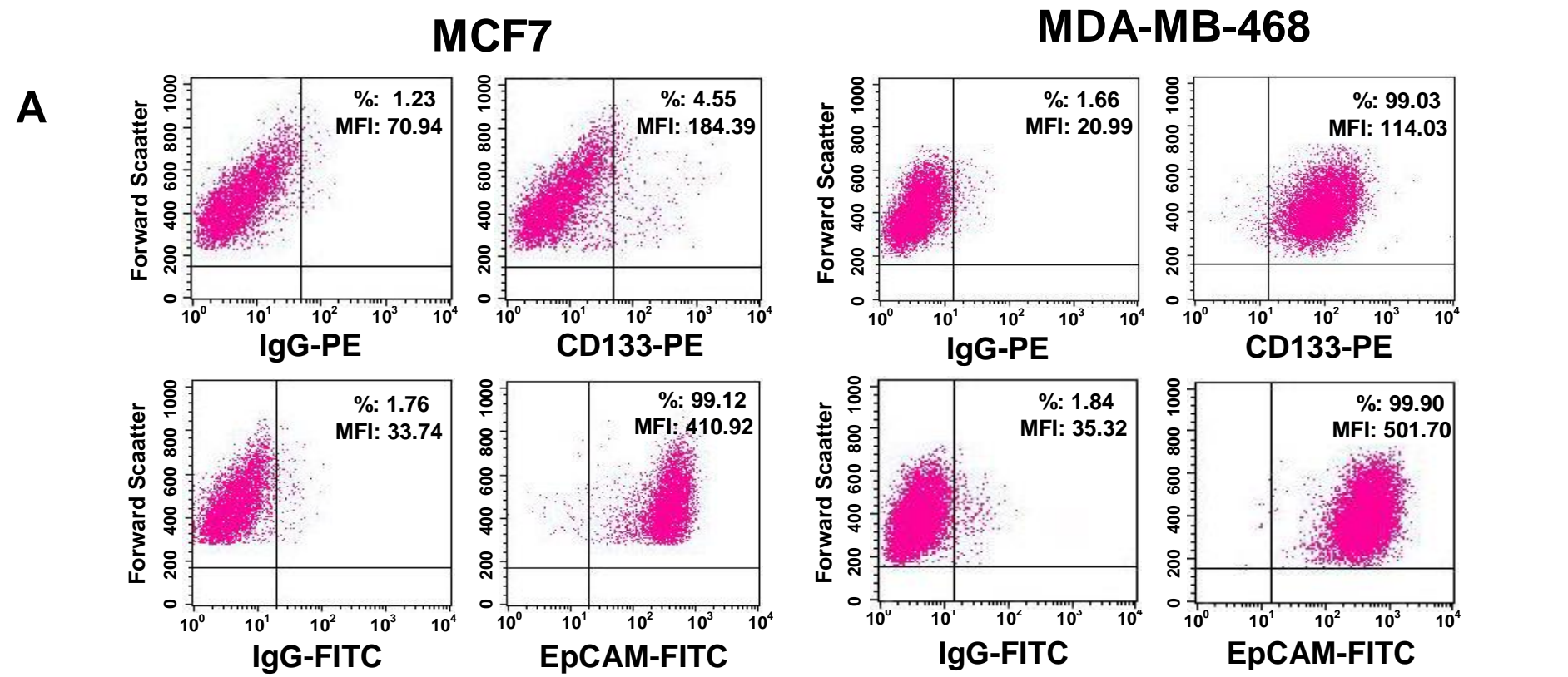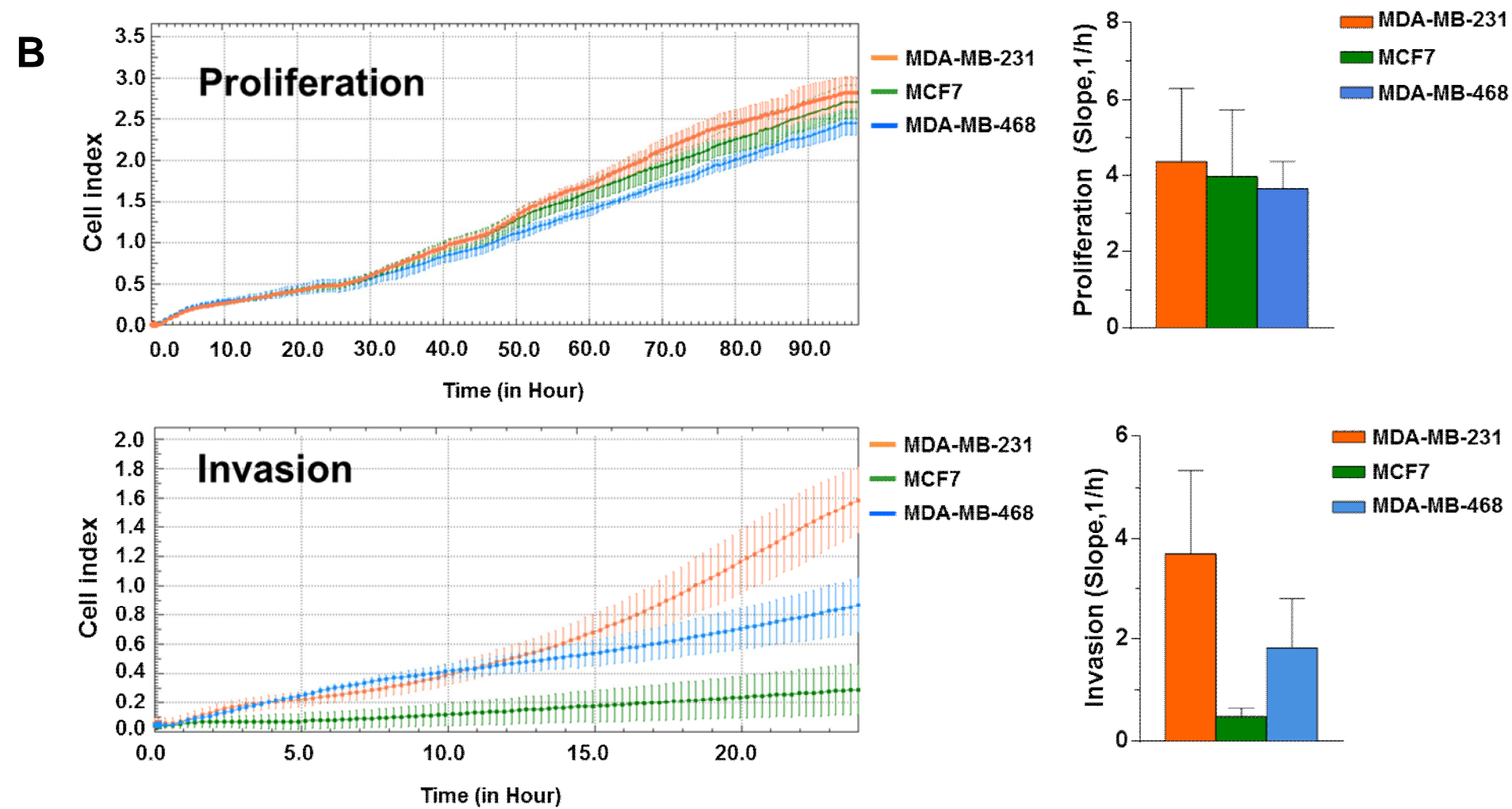

Supplement: Additional file 1: Figure S1. — Surface CD133 and EpCAM, proliferation and invasiveness in breast derived cell lines. In A, representative cytofluorimetrical evaluation of CD133 and EpCAM surface levels in MCF7 and MDA-MB-468 cells after labelling with a PE-conjugated anti-CD133 antibody or with a FITC-conjugated anti-EpCAM antibody. The staining with isotype matched antibodies (IgG) is used as a control. The expression of each antigen is shown on a biparametric dot plot and the percentage and MFI of positive cells are indicated at the upper right of each panel. In B, MDA-MB-231, MCF7 and MDA-MB-468 cells were subjected to dynamic monitoring of proliferation and invasion through Matrigel using the xCELLigence RTCA system. Cell Index (CI) is reported and error bars indicate ±SD. The correspondent Slope analysis, that describes the steepness, incline, gradient, and changing rate of the CI curves over time, is shown on the right. The data were collected from three separate experiments (PDF 364 kb) [file 12885_2017_3592_MOESM1_ESM.pdf]
